# 1-[3-(4-Methoxy­phen­yl)-6-methyl-1,6-dihydro-1,2,4,5-tetra­zin-1-yl]propanone

**DOI:** 10.1107/S1600536810011165

**Published:** 2010-03-27

**Authors:** Zhen-zhen Yang, Feng Xu, Hong-yun Chen

**Affiliations:** a Department of Biological and Chemical Engineering, Taizhou Vocational and Technical college, Taizhou 318000, People’s Republic of China

## Abstract

In the title compound, C_13_H_16_N_4_O_2_, the central tetra­zine ring adopts an unsymmetrical boat conformation with the two C atoms as flagpoles. This compound can be considered as having homoaromaticity.

## Related literature

For the biological activity of 1,2,4,5-tetra­zine derivatives, see: Sauer (1996 ▶). For related structures, see: Jennison *et al.* (1986 ▶); Stam *et al.* (1982 ▶); Xu *et al.* (2010 ▶). For the structure–activity relationships of 1,6-dihydro-1,2,4,5-tetra­zine derivatives, see: Hu *et al.* (2004 ▶, 2005 ▶).
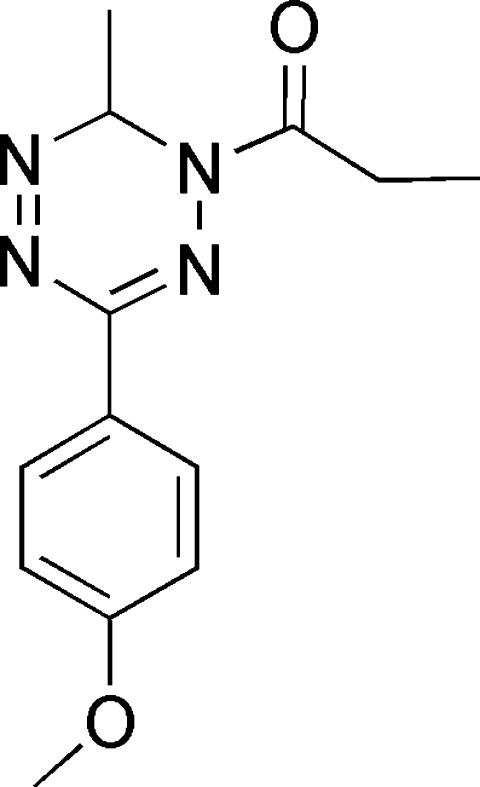


## Experimental

### 

#### Crystal data

C_13_H_16_N_4_O_2_
*M* *_r_* = 260.30
Triclinic, 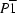

*a* = 8.345 (2) Å
*b* = 8.4898 (19) Å
*c* = 10.245 (3) Å
α = 113.232 (6)°
β = 99.820 (15)°
γ = 93.268 (11)°
*V* = 651.0 (3) Å^3^
*Z* = 2
Mo *K*α radiation
μ = 0.09 mm^−1^
*T* = 93 K
0.50 × 0.37 × 0.23 mm

#### Data collection

Rigaku AFC10/Saturn724+ diffractometer
6410 measured reflections
2920 independent reflections
2207 reflections with *I* > 2σ(*I*)
*R*_int_ = 0.022

#### Refinement

*R*[*F* ^2^ > 2σ(*F* ^2^)] = 0.036
*wR*(*F* ^2^) = 0.081
*S* = 1.00
2920 reflections
175 parameters
H-atom parameters constrained
Δρ_max_ = 0.32 e Å^−3^
Δρ_min_ = −0.21 e Å^−3^

### 

Data collection: *CrystalClear* (Rigaku/MSC, 2008 ▶); cell refinement: *CrystalClear*; data reduction: *CrystalClear*; program(s) used to solve structure: *SHELXS97* (Sheldrick, 2008 ▶); program(s) used to refine structure: *SHELXL97* (Sheldrick, 2008 ▶); molecular graphics: *SHELXTL* (Sheldrick, 2008 ▶); software used to prepare material for publication: *SHELXTL*.

## Supplementary Material

Crystal structure: contains datablocks global, I. DOI: 10.1107/S1600536810011165/ci5067sup1.cif

Structure factors: contains datablocks I. DOI: 10.1107/S1600536810011165/ci5067Isup2.hkl

Additional supplementary materials:  crystallographic information; 3D view; checkCIF report

## Figures and Tables

**Fig. 1.**
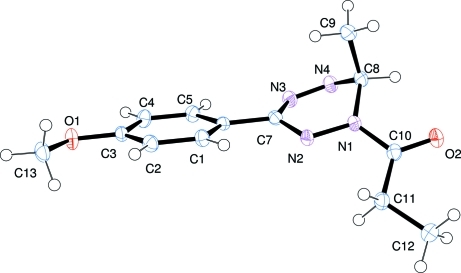
The molecular structure of the title compound, showing 50% probability displacement ellipsoids and the atomic numbering.

## supplementary crystallographic information

### Comment

1,2,4,5-Tetrazine derivatives have high potential for biological activity, possessing a wide spectrum of antiviral and antitumor properties. They have been widely used in pesticides and herbicides (Sauer, 1996). Dihydro-1,2,4,5- tetrazine has four isomers, namely 1,2-, 1,4-, 1,6- and 3,6-dihydro-1,2,4,5- tetrazines. The 1,6-dihydro structures (Stam *et al.*, 1982; Jennison *et al.*, 1986) were found, by X-ray diffraction, to be homoaromatic. In continuation of our work on the structure-activity relationship of 1,6-dihydro-1,2,4,5-tetrazine derivatives (Hu *et al.*, 2004,2005), we report here the crystal structure of the title compound (I) (Fig. 1).

In the tetrazine ring, atoms N1, N2, N3 and N4 are coplanar, while atoms C7 and C8 deviate from the plane by 0.239 (2) and 0.595 (2) Å, respectively. The N2/C7/N3 and N1/C8/N4 planes make dihedral angles of 21.0 (2)° and 42.2 (1)°, respectively, with the N1/N2/ N3/N4 plane, *i.e.* the tetrazine ring adopts an unsymmetrical boat conformation. The benzene ring make dihedral angle of 16.7 (1)° with the N1/N2/N3/N4 plane. Atom N1 is almost *sp*^2^ hybridized due to the angles around it add up to 360.0 (2)°. In keeping with similar situations in 3-phenyl-6-ethyl-1,6-dihydro- 1,2,4,5-tetrazine (Stam *et al.*, 1982), *N*-(2-methylphenyl)-3-phenyl-6-methyl-1,6-dihydro-1,2,4,5-tetrazine (Xu *et al.*, 2010) and 1-acetyl-3,6-dimethyl-1,2,4,5-tetrazine (Jennison *et al.*, 1986), it can be considered that the molecule is homoaromatic.

### Experimental

3-(4-Methoxyphenyl)-6-methyl-1,6-dihydro-1,2,4,5-tetrazine (3.0 mmol), chloroform (10 ml) and pyridine (0.25 ml,3.1 mmol) were mixed. Propionyl chloride(3.0 mmol) in chloroform (10 ml) was added dropwise with stirring at room temperature. After the starting, 1,6-dihydro-1,2,4,5-tetrazine was completely consumed (the reaction courses was monitored by TLC, dichloromethane system), evaporation of the chloroform, crude 1-propionyl-3-(4-methoxyphenyl)-6-methyl- 1,6-dihydro-1,2,4,5-tetrazine was obtained and purified by preparative thin-layer chromatography over silica gel GF254(2 mm) (dichloromethane-petroleum ether, 1:1). The solution of the compound in anhydrous ethanol was concentrated gradually at room temperature to afford single crystals, which was suitable for X-ray diffraction (m.p. 340–342 K).^1^H NMR (CDCl_3_) δ p.p.m.: 8.10 (d,2*H*, J = 8.8 Hz), 7.03 (d,2*H*, J = 8.8 Hz), 6.84 (q,1*H*, J = 6.4 Hz), 3.89 (s,3*H*), 2.95–3.05(m,1*H*,CH_2_), 2.72–2.85(m,1*H*,CH_2_),1.20(t,3*H*, J = 7.6 Hz), 1.04 (d,3*H*, J = 6.4 Hz).

### Refinement

H atoms were placed in calculated positions with N—H = 0.86 Å, C-H = 0.93 (aromatic) and 0.96 Å (methyl), and refined in riding model, with *U*_iso_(H) = 1.2*U*_eq_(C,N) and 1.5*U*_eq_(C_methyl_).

### Crystal data

**Table:**

C_13_H_16_N_4_O_2_	*Z* = 2
*M**_r_* = 260.30	*F*(000) = 276
Triclinic, *P*1	*D*_x_ = 1.328 Mg m^−^^3^
Hall symbol: -P 1	Mo *K*α radiation, λ = 0.71073 Å
*a* = 8.345 (2) Å	Cell parameters from 1846 reflections
*b* = 8.4898 (19) Å	θ = 3.4–27.5°
*c* = 10.245 (3) Å	µ = 0.09 mm^−^^1^
α = 113.232 (6)°	*T* = 93 K
β = 99.820 (15)°	Prism, red
γ = 93.268 (11)°	0.50 × 0.37 × 0.23 mm
*V* = 651.0 (3) Å^3^	

### Data collection

**Table:**

Rigaku AFC10/Saturn724+ diffractometer	2207 reflections with *I* > 2σ(*I*)
Radiation source: Rotating Anode	*R*_int_ = 0.022
graphite	θ_max_ = 27.5°, θ_min_ = 3.4°
Detector resolution: 28.5714 pixels mm^-1^	*h* = −10→10
φ and ω scans	*k* = −11→11
6410 measured reflections	*l* = −13→12
2920 independent reflections	

### Refinement

**Table:**

Refinement on *F*^2^	Primary atom site location: structure-invariant direct methods
Least-squares matrix: full	Secondary atom site location: difference Fourier map
*R*[*F*^2^ > 2σ(*F*^2^)] = 0.036	Hydrogen site location: inferred from neighbouring sites
*wR*(*F*^2^) = 0.081	H-atom parameters constrained
*S* = 1.00	*w* = 1/[σ^2^(*F*_o_^2^) + (0.0282*P*)^2^ + 0.16*P*] where *P* = (*F*_o_^2^ + 2*F*_c_^2^)/3
2920 reflections	(Δ/σ)_max_ = 0.001
175 parameters	Δρ_max_ = 0.32 e Å^−^^3^
0 restraints	Δρ_min_ = −0.21 e Å^−^^3^

### Special details

**Table:**

Geometry. All esds (except the esd in the dihedral angle between two l.s. planes) are estimated using the full covariance matrix. The cell esds are taken into account individually in the estimation of esds in distances, angles and torsion angles; correlations between esds in cell parameters are only used when they are defined by crystal symmetry. An approximate (isotropic) treatment of cell esds is used for estimating esds involving l.s. planes.
Refinement. Refinement of *F*^2^ against ALL reflections. The weighted *R*-factor wR and goodness of fit *S* are based on *F*^2^, conventional *R*-factors *R* are based on *F*, with *F* set to zero for negative *F*^2^. The threshold expression of *F*^2^ > σ(*F*^2^) is used only for calculating *R*-factors(gt) etc. and is not relevant to the choice of reflections for refinement. *R*-factors based on *F*^2^ are statistically about twice as large as those based on *F*, and *R*- factors based on ALL data will be even larger.

### Fractional atomic coordinates and isotropic or equivalent isotropic displacement parameters (Å^2^)

**Table:**

	*x*	*y*	*z*	*U*_iso_*/*U*_eq_	
O1	1.03435 (11)	0.17376 (12)	0.48191 (9)	0.0240 (2)	
O2	0.16763 (10)	0.73207 (11)	0.11227 (10)	0.0240 (2)	
N1	0.33360 (12)	0.55086 (13)	0.15664 (11)	0.0189 (2)	
N2	0.47200 (12)	0.53199 (13)	0.23910 (11)	0.0186 (2)	
N3	0.47565 (13)	0.27926 (13)	0.02813 (11)	0.0209 (2)	
N4	0.34190 (13)	0.29167 (14)	−0.04264 (11)	0.0224 (2)	
C1	0.69924 (15)	0.41266 (16)	0.41273 (13)	0.0193 (3)	
H1	0.6425	0.5041	0.4631	0.023*	
C2	0.82340 (15)	0.36341 (16)	0.49081 (13)	0.0198 (3)	
H2	0.8512	0.4207	0.5938	0.024*	
C3	0.90723 (14)	0.22979 (16)	0.41785 (13)	0.0179 (3)	
C4	0.86288 (15)	0.14452 (16)	0.26693 (13)	0.0213 (3)	
H4	0.9183	0.0516	0.2169	0.026*	
C5	0.73919 (15)	0.19411 (16)	0.18974 (13)	0.0198 (3)	
H5	0.7102	0.1351	0.0869	0.024*	
C6	0.65589 (14)	0.33049 (15)	0.26145 (13)	0.0166 (3)	
C7	0.52322 (15)	0.38258 (16)	0.18025 (13)	0.0174 (3)	
C8	0.23071 (15)	0.39564 (16)	0.04682 (13)	0.0197 (3)	
H8	0.1475	0.4295	−0.0163	0.024*	
C9	0.14232 (16)	0.29206 (17)	0.11103 (14)	0.0229 (3)	
H9A	0.2213	0.2738	0.1846	0.027*	
H9B	0.0908	0.1798	0.0339	0.027*	
H9C	0.0577	0.3556	0.1561	0.027*	
C10	0.29499 (15)	0.71638 (16)	0.18095 (13)	0.0188 (3)	
C11	0.41530 (15)	0.86447 (16)	0.29536 (13)	0.0211 (3)	
H11A	0.5256	0.8546	0.2720	0.025*	
H11B	0.4215	0.8583	0.3906	0.025*	
C12	0.36603 (17)	1.03756 (17)	0.30564 (15)	0.0267 (3)	
H12A	0.3573	1.0429	0.2109	0.032*	
H12B	0.4492	1.1311	0.3782	0.032*	
H12C	0.2598	1.0506	0.3345	0.032*	
C13	1.07827 (16)	0.24669 (18)	0.63702 (14)	0.0246 (3)	
H13A	1.1083	0.3722	0.6737	0.030*	
H13B	1.1719	0.1957	0.6680	0.030*	
H13C	0.9848	0.2222	0.6757	0.030*	

### Atomic displacement parameters (Å^2^)

**Table:**

	*U*^11^	*U*^22^	*U*^33^	*U*^12^	*U*^13^	*U*^23^
O1	0.0227 (5)	0.0325 (5)	0.0196 (5)	0.0115 (4)	0.0026 (4)	0.0134 (4)
O2	0.0192 (5)	0.0280 (5)	0.0261 (5)	0.0064 (4)	0.0008 (4)	0.0134 (4)
N1	0.0169 (5)	0.0206 (5)	0.0185 (5)	0.0019 (4)	−0.0015 (4)	0.0094 (4)
N2	0.0166 (5)	0.0217 (5)	0.0192 (5)	0.0028 (4)	0.0004 (4)	0.0114 (4)
N3	0.0206 (6)	0.0244 (6)	0.0174 (5)	0.0031 (4)	0.0013 (4)	0.0094 (5)
N4	0.0210 (6)	0.0256 (6)	0.0195 (5)	0.0030 (5)	0.0004 (5)	0.0096 (5)
C1	0.0184 (6)	0.0202 (6)	0.0187 (6)	0.0047 (5)	0.0037 (5)	0.0073 (5)
C2	0.0205 (6)	0.0224 (6)	0.0151 (6)	0.0019 (5)	0.0014 (5)	0.0073 (5)
C3	0.0147 (6)	0.0219 (6)	0.0200 (6)	0.0027 (5)	0.0020 (5)	0.0123 (5)
C4	0.0239 (7)	0.0220 (6)	0.0199 (6)	0.0088 (5)	0.0065 (5)	0.0091 (5)
C5	0.0227 (7)	0.0210 (6)	0.0144 (6)	0.0033 (5)	0.0022 (5)	0.0066 (5)
C6	0.0147 (6)	0.0182 (6)	0.0181 (6)	0.0001 (5)	0.0017 (5)	0.0096 (5)
C7	0.0157 (6)	0.0207 (6)	0.0169 (6)	0.0007 (5)	0.0026 (5)	0.0094 (5)
C8	0.0173 (6)	0.0218 (6)	0.0180 (6)	0.0017 (5)	−0.0015 (5)	0.0083 (5)
C9	0.0200 (6)	0.0238 (7)	0.0237 (7)	0.0003 (5)	0.0008 (5)	0.0103 (5)
C10	0.0188 (6)	0.0232 (6)	0.0184 (6)	0.0053 (5)	0.0057 (5)	0.0118 (5)
C11	0.0212 (7)	0.0218 (7)	0.0209 (7)	0.0048 (5)	0.0024 (5)	0.0101 (5)
C12	0.0261 (7)	0.0213 (7)	0.0332 (8)	0.0049 (5)	0.0047 (6)	0.0120 (6)
C13	0.0222 (7)	0.0329 (7)	0.0200 (7)	0.0043 (6)	−0.0006 (5)	0.0141 (6)

### Geometric parameters (Å, °)

**Table:**

O1—C3	1.3596 (14)	C5—C6	1.3995 (17)
O1—C13	1.4291 (15)	C5—H5	0.95
O2—C10	1.2155 (14)	C6—C7	1.4647 (16)
N1—N2	1.3669 (14)	C8—C9	1.5160 (17)
N1—C10	1.3941 (16)	C8—H8	1.00
N1—C8	1.4531 (15)	C9—H9A	0.98
N2—C7	1.3044 (16)	C9—H9B	0.98
N3—N4	1.2571 (14)	C9—H9C	0.98
N3—C7	1.4236 (16)	C10—C11	1.5015 (17)
N4—C8	1.4917 (16)	C11—C12	1.5169 (17)
C1—C2	1.3852 (17)	C11—H11A	0.99
C1—C6	1.3952 (17)	C11—H11B	0.99
C1—H1	0.95	C12—H12A	0.98
C2—C3	1.3911 (17)	C12—H12B	0.98
C2—H2	0.95	C12—H12C	0.98
C3—C4	1.3939 (17)	C13—H13A	0.98
C4—C5	1.3791 (17)	C13—H13B	0.98
C4—H4	0.95	C13—H13C	0.98
			
C3—O1—C13	118.23 (10)	N1—C8—H8	108.9
N2—N1—C10	119.47 (10)	N4—C8—H8	108.9
N2—N1—C8	118.20 (10)	C9—C8—H8	108.9
C10—N1—C8	122.32 (10)	C8—C9—H9A	109.5
C7—N2—N1	113.70 (10)	C8—C9—H9B	109.5
N4—N3—C7	120.02 (11)	H9A—C9—H9B	109.5
N3—N4—C8	115.25 (10)	C8—C9—H9C	109.5
C2—C1—C6	121.25 (12)	H9A—C9—H9C	109.5
C2—C1—H1	119.4	H9B—C9—H9C	109.5
C6—C1—H1	119.4	O2—C10—N1	119.15 (11)
C1—C2—C3	119.76 (11)	O2—C10—C11	124.57 (12)
C1—C2—H2	120.1	N1—C10—C11	116.27 (10)
C3—C2—H2	120.1	C10—C11—C12	111.53 (11)
O1—C3—C2	125.16 (11)	C10—C11—H11A	109.3
O1—C3—C4	115.33 (11)	C12—C11—H11A	109.3
C2—C3—C4	119.50 (11)	C10—C11—H11B	109.3
C5—C4—C3	120.48 (12)	C12—C11—H11B	109.3
C5—C4—H4	119.8	H11A—C11—H11B	108.0
C3—C4—H4	119.8	C11—C12—H12A	109.5
C4—C5—C6	120.64 (11)	C11—C12—H12B	109.5
C4—C5—H5	119.7	H12A—C12—H12B	109.5
C6—C5—H5	119.7	C11—C12—H12C	109.5
C1—C6—C5	118.35 (11)	H12A—C12—H12C	109.5
C1—C6—C7	120.72 (11)	H12B—C12—H12C	109.5
C5—C6—C7	120.91 (11)	O1—C13—H13A	109.5
N2—C7—N3	121.12 (11)	O1—C13—H13B	109.5
N2—C7—C6	121.13 (11)	H13A—C13—H13B	109.5
N3—C7—C6	116.68 (11)	O1—C13—H13C	109.5
N1—C8—N4	106.07 (9)	H13A—C13—H13C	109.5
N1—C8—C9	112.98 (10)	H13B—C13—H13C	109.5
N4—C8—C9	110.99 (10)		
			
C10—N1—N2—C7	−159.83 (10)	N4—N3—C7—C6	164.47 (10)
C8—N1—N2—C7	20.73 (14)	C1—C6—C7—N2	18.43 (17)
C7—N3—N4—C8	−9.80 (16)	C5—C6—C7—N2	−163.36 (11)
C6—C1—C2—C3	0.09 (18)	C1—C6—C7—N3	−173.30 (11)
C13—O1—C3—C2	5.43 (17)	C5—C6—C7—N3	4.91 (16)
C13—O1—C3—C4	−175.44 (11)	N2—N1—C8—N4	−52.33 (13)
C1—C2—C3—O1	177.78 (11)	C10—N1—C8—N4	128.25 (11)
C1—C2—C3—C4	−1.32 (18)	N2—N1—C8—C9	69.47 (13)
O1—C3—C4—C5	−177.85 (11)	C10—N1—C8—C9	−109.95 (13)
C2—C3—C4—C5	1.33 (18)	N3—N4—C8—N1	45.04 (13)
C3—C4—C5—C6	−0.12 (18)	N3—N4—C8—C9	−78.02 (13)
C2—C1—C6—C5	1.10 (17)	N2—N1—C10—O2	−176.38 (11)
C2—C1—C6—C7	179.36 (11)	C8—N1—C10—O2	3.04 (17)
C4—C5—C6—C1	−1.09 (17)	N2—N1—C10—C11	2.16 (16)
C4—C5—C6—C7	−179.34 (11)	C8—N1—C10—C11	−178.43 (10)
N1—N2—C7—N3	20.73 (16)	O2—C10—C11—C12	−5.10 (17)
N1—N2—C7—C6	−171.53 (10)	N1—C10—C11—C12	176.46 (11)
N4—N3—C7—N2	−27.26 (17)		
